# The perception of allergen-specific immunotherapy among pediatricians in the primary care setting

**DOI:** 10.1186/s12948-015-0021-0

**Published:** 2015-07-24

**Authors:** Massimo Landi, Paolo Meglio, Ermanno Praitano, Carlo Lombardi, Giovanni Passalacqua, Giorgio Walter Canonica

**Affiliations:** Italian Federation of Pediatric Primary Care, Turin, Italy; Italian Federation of Pediatric Primary Care, Rome, Italy; Italian Federation of Pediatric Primary Care, Bari, Italy; Allergy Unit, Department of Internal Medicine, Sant’Orsola-Poliambulanza Hospital, Brescia, Italy; Allergy and Respiratory Diseases, DIMI, IRCCS San Martino Hospital-IST-University of Genoa, Pad.Maragliano, L.go R Benzi 10, 16133 Genoa, Italy

**Keywords:** Primary care pediatrician, Children, Allergic respiratory disease, Allergen immunotherapy

## Abstract

**Background:**

Allergic respiratory diseases are constantly increasing in prevalence. Allergen Immunotherapy (AIT) represent a valuable therapeutic tool as symptomatic and preventative approach, expecially in children. In Italy, primary care pediatricians (PCP) represent the first-line contact and interface for prescription, use and management of AIT. We attempted to evaluate the perception of AIT practice among PCP.

**Methods:**

A questionnaire was built-up, based on literature, guidelines and with the contribution of pediatricians. The questionnaire, including 12 items, was e-mailed to 180 PCP, randomly chosen from mailing lists. The questionnaire explored the personal perception of AIT, the comparison between subcutaneous and sublingual AIT and the overall awareness about the treatment.

**Results:**

130 questionnaires were eligible for analysis. There was a satisfactory knowledge of the characteristics of AIT, its aims and limits, although the positioning of the treatment in guidelines was insufficiently known. Overall, the prescription of AIT made by other specialists was accepted and agreed (78 %). The majority of pediatricians felt that a more intense divulgation and information about AIT would be needed (90 %).

**Conclusion:**

AIT is in general well known and accepted among PCP, although a more intense divulgation effort is required.

## Background

The prevalence of allergic respiratory diseases (rhinitis and asthma) constantly increased in the second half of the last century. Nowadays, although the prevalence of asthma seemed to plateau, that of rhinitis is still increasing. This fact may be attributable to different factors, including the “westernization” of lifestyle, the reduction of infectious diseases, and the increased allergenic burden linked to climate changes [1, 2]. Concerning Italy, the SIDRIA survey [3], conducted in 1994–95 and 2002, confirmed the plateau of asthma prevalence (about 10 % in both children and adolescents), and the significant increase of rhinitis (from 6 to 9 % in children and from 14 to 17 % in adolescents). In this survey, no relevant difference could be seen in the geographical distribution of respiratory allergies, with a slightly higher occurrence of the symptom cough in large urban agglomerates with respect to rural areas.

In the pediatric age range, allergic diseases represent a special problem, with specific aspects, that include their possible evolution (allergic march) [4–6], the problems related to the long-term pharmacotherapy, the compliance (which is in charge of caregivers), the objective difficulties in correctly deliver inhaled drugs. In addition, the quality of life of the children themselves and of their parents (drug treatment, emergency unit visits, impaired school performance and absenteeism), is usually affected [7, 8]. Thus, an early and correct diagnosis and an adequate therapeutic management of allergic respiratory diseases in children are strongly desirable [9].

Many clinical trials and meta-analyses have convincingly demonstrated that allergen immunotherapy (AIT) is effective in reducing symptoms and drug consumption, with a consequent improvement of the overall quality of life. More importantly, there is evidence that AIT can modify the natural history of allergic disease, for instance preventing the onset of asthma in patients with allergic rhinitis and/or maintaining a long-lasting effect [10]. The introduction of the sublingual route of administration (SLIT) in clinical practice represented an important step forward, of particular relevance in the pediatric age [11]. In this case, the good safety profile (very low risk of severe adverse events), the convenience and the management at home, allowed to expand the indications. In fact, more and more often SLIT is prescribed to children below the age of 5 years [12, 13].

Pediatricians are the first-line specialists who afford the problems related to respiratory allergy, to the prescription and to management of AIT. In Italy, the figure of the “primary care” pediatrician, makes the situation even more peculiar, since those healthcare specialists remain a solid and constant contact with children and families. Based on these premises we performed a questionnaire-based survey among Italian primary care pediatricians to assess their knowledge and information about the use of AIT.

## Methods

A 12-item questionnaire was built up, based on the current literature, Position Papers [14–16], expert opinions, and the contribution of numerous primary care pediatricians (Table 1). The questionnaire, involving prescription attitudes, follow-up of patients, awareness about the treatment, had multiple choice options. The questionnaire was e-mailed to primary care pediatricians, randomly chosen from the mailing lists of participants to meetings over the whole Italian territory. The study was observational and cross sectional, therefore only required notification to the local Ethic Committees, according to the Italian laws. The participating pediatricians were required to refer only to those patients for whom the diagnosis of respiratory allergy, and the prescription of AIT were clearly established, possibly made by pediatric allergists/pulmonologists.

**Table 1:** The questionnaire and percentage of responses

N	Item	Percent
1	AITs is :	
	a. a treatment to contrast the allergen-related symptoms in general	32.7
	b. a target organ treatment (rhinitis/asthma)	10
	c. an alternative to pharmacotherapy	1.6
	d. a complementary adjunct when pharmacotherapy is not sufficient to control symptoms	55.7
2	Do you consider AIT useful/effective in allergic rhinitis?	
	a. Yes, always.	8.6
	b. Yes, in most cases	44.5
	c. Only in selected cases	34.4
	d. No	12.5
3	Do you consider AIT useful/effective in allergic bronchial asthma?	
	a. Yes, always.	17.3
	b. Yes, in most cases	49.6
	c. Only in selected cases	31.5
	d. No	1.6
4	According to your clinical practice, does AIT result in a pharmaco-economic saving?	
	a. Yes, always.	37.5
	b. Only in selected cases	49.2
	c. No	4.7
	d. No opinion	8.6
5	Do you think that AIT adds clinical beneficial effects to standard treatment?	
	a. Yes, always.	47.6
	b. Only in selected cases	43.7
	c. No	6.3
	d. No opinion	2.4
6	Based on your experience/literature, do you think that AIT can prevent the onset of new sensitizations?	
	a. Yes, always.	24.2
	b. Only in selected cases	33.6
	c. No	26.6
	d. No opinion	15.6
7	Based on your experience/literature, do you think that AIT can modify the progression of disease?	
	a. Yes, always.	30.7
	b. Only in selected cases	59.8
	c. No	9.5
	d. No opinion	0
8	Based on your experience and literature, do you think that SCIT and SLIT are equivalent for safety?	
	a. Yes	22.8
	b. NO, SCIT is better than SLIT	12.6
	c. NO, SLIT is better than SCIT	56.7
	d. No opinion	7.9
9	When a children is prescribed with AIT by the specialist, which is your attitude?	
	a. Agree at all	78.4
	b. I require more details	18.4
	c. Disagree	1.6
	d. Indifferent	1.6
10	Based on your experience and literature, do you think that SCIT and SLIT are equally effective?	
	a. Yes	45.3
	b. No, SCIT is better than SLIT	23.4
	c. No, SLIT is better than SCIT	10.2
	d. Do not know	21.1
11	Do you think that more interest should be deserved to AIT in the congresses you usually attend?	
	a. Yes	92.9
	b. No	7.1
12	In international guidelines for rhinitis (ARIA) and asthma (GINA) is AIT mentioned?	
	a. Yes, in both	55.4
	b. No	24.8
	c. Only in ARIA guidelines	8.3
	d. Only in GINA guidelines	11.6

## Results

The questionnaire was e-mailed to 180 primary care pediatricians, of whom 140 responded. Out of the 140 returned questionnaires, 130 were eligible for analysis since correctly and completely filled. The respondent pediatrician had an age of 55 ± 8.3 years, and 50 of them were male. All of them were obviously employed in the primary care service. On average, each pediatrician was in charge of about 1,000 children (1050 ± 245). The pediatricians resulted to be distributed as follows: northern (31 %) central (24 %) and southern (46 %) Italy, with no difference in gender and age distribution among the three geographical regions. The results are reported in Table 1. As per responses, there was a satisfactory knowledge of the characteristics of AIT, its aims and limits. Overall, the prescription of AIT made by other specialists (pediatric allergist/pulmonologist) was accepted and agreed (78 %). Of note, the majority of pediatricians felt that a more intense divulgation and information about AIT would be needed (90 %). There was no difference in the distribution of the answers according to gender (not shown), and a comparative analysis according to age range could not be made due to the important skew towards older ages (80 % of respondents over 45 years of age and 94 % over 35 years). Also, no geographical-related difference in responses could be detected among the area of residency, as summarized in Table 2.

**Table 2:** Percentage of positive responses to each item according to the geographical location

Item^a^	North	Center	South	Item^a^	North	Center	South
	(N = 38)	(N = 33)	(N = 59)		(N = 38)	(N = 33)	(N = 59)
	%	%	%		%	%	%
1 a	37	38	28	7 a	30	28	31
1 b	11	7	10	7 b	60	64	56
1 c	0	0	5	7 c	10	7	13
1 d	52	55	57	7 d	0	0	0
2 a	7	9	9	8 a	18	21	24
2 b	39	44	54	8 b	11	20	12
2 c	41	36	28	8 c	60	59	54
2 d	13	11	9	8 d	11	10	10
23 a	14	23	18	9 a	76	78	78
3 b	55	32	55	9 b	20	19	16
3 c	31	45	23	9 c	2	0	4
3 d	0	0	4	9 d	2	3	2
4 a	43	41	30	10 a	48	51	42
4 b	45	45	56	10 b	16	24	27
4 c	4	7	4	10 c	16	6	8
4 d	8	7	10	10 d	20	22	23
5 a	53	61	46	11 a	93	96	95
5 b	39	30	48	11 b	7	4	5
5 c	5	9	6				
5 d	3	0	0				
6 a	24	31	22	12 a	45	45	41
6 b	38	38	36	12 b	23	18	26
6 c	21	24	24	12 c	12	18	20
6 d	17	7	12	12 d	20	19	13

No significant difference among the 3 regions was detectable for all items

^**a**^For the description of each item refer to Table 1

## Discussion

As mentioned above, AIT is the only allergen-oriented therapy, and acts as a disease-modifying treatment. Thus, AIT can not only modify symptoms in the short-medium term period, but can change the progression of the disorder. The disease-modifying effect can be seen as the reduction of the risk of asthma onset in children with rhinitis, and as the persistence of the clinical benefit for several years after the discontinuation [10, 17–19]. These facts assume a special relevance in the pediatric age, when the plasticity and modulability of the immune system are maximal, and when the preventative effects can be reasonably expected.

In Italy, children are followed-up by the institutional primary care pediatrician, an almost unique figure all over the world (the equivalent of the general practitioner for adults), who faces daily allergic diseases and their management as first-line referral. AIT is usually prescribed by pediatric allergists (a subspecialty in Italy), who diagnose the disease and chose the most appropriate AIT, then leaving it in the hands of the primary care pediatricians. In such a setting, we considered of primary importance to know which is the perception of and the attitude toward AIT within general pediatricians. This was explored by a simple questionnaire developed by a panel of experts, on the basis of the literature, and agreed with a representative number of primary care colleagues. According to the results, the overall knowledge on the specific argument seems to be satisfactory. Notably, about 50 % of pediatricians still believe that AIT is an add-on therapy to be used when pharmacotherapy fails. Another important aspect is that only one half of the interviewed pediatricians are aware of the fact that AIT is mentioned in the major guidelines. Finally, and probably according to the mentioned responses, the majority of primary care pediatrician agree on the fact that a more intense divulgation effort on the specific argument is worthwhile. The main limitation of this study stands in the questionnaire-based method, with the questionnaire prepared by a restricted group of experts. Nonetheless, it has to be considered that, to obtain a satisfactory response, the questionnaire itself had to be kept as simple and as short as possible. In addition, since no explanation or information on AIT was provided when the questionnaires were mailed, we can assume that the responses truly reflect the reality. Finally it is not possible to compare our results with other similar, since the primary care pediatircian is a professional figure that is present only in our Country (and very few others), and no survey in this sense has been attempted in the past.

## Conclusion

As a general consideration, the results herein reported are overall in agreement with those described for Italian general practitioners and chest physicians, who were previously interviewed using similar questionnaires [20, 21]. This facts indirectly testifies that there is an increasing awareness about AIT among physicians, as repeatedly auspicated [22].
